# Endoluminal Vacuum Therapy as a Salvage Procedure for Difficult Anastomotic Leak Post Roux-en-Y Gastric Bypass

**DOI:** 10.7759/cureus.59313

**Published:** 2024-04-29

**Authors:** Pranav Balakrishnan, Armein Rahimpour, Semeret T Munie, Darren B Nease

**Affiliations:** 1 General Surgery, Marshall University Joan C. Edwards School of Medicine, Huntington, USA; 2 Bariatric Surgery, Marshall University Joan C. Edwards School of Medicine, Huntington, USA

**Keywords:** robotic bariatric surgery, revisional bariatric surgery, gastric sleeve leak, advanced endoscopy, endoluminal vacuum, robotic gastric bypass, roux-en-y gastric bypass (rygb), gastric sleeve, bariatric surgery complications

## Abstract

We present a case of a woman in her 60s, with a history of a gastric sleeve resection, over 50% excess body weight loss, and subsequent severe gastroesophageal reflux disease refractory to maximal medical therapy, who underwent a conversion of a sleeve gastrectomy to a Roux-en-Y gastric bypass with hiatal hernia repair. On postoperative day five, she was evaluated at our emergency department for vomiting and inability to tolerate oral intake. Imaging revealed a large retrocardiac hiatal hernia and extraluminal contrast extravasation.

She was taken to the operating room after resuscitation, where the gastric pouch and roux limb were found to have significant edema with recurrence of the hernia. This was able to be reduced and a frank perforation was found at the posterior aspect of the anastomosis. A covered metal stent was placed by the gastroenterologist and drains were left in place.

In the ICU, nasojejunal feeds were stopped given suspicion of backflow with persistent leak. A decision was made to remove the stent and place an endoluminal vacuum (endoscopic vacuum-assisted wound closure [EVAC]). After three subsequent vacuum-sponge changes, the perforation was found to have healed. Patient was tolerating a diet on discharge.

This case is an example of a complication where a multidisciplinary approach to a difficult leak resulted in recovery with the use of EVAC. We believe this is a valuable tool to have in our armamentarium for difficult-to-manage leaks.

## Introduction

Anastomotic leaks are a dreaded complication following bariatric surgery [1]. There has been a marked decrease in the anastomotic leak rate compared to data drawn from multiple different case series ranging from 0.1% to 8.3% following laparoscopic gastric bypass (LGBP) [1-3]. Endoscopic vacuum-assisted wound closure (EVAC) consists of endoscopic debridement and washout, measurement of the cavity resulting from the leak, placement of endosponge, and connecting it to continuous suction at 175 mm Hg via a nasogastric tube. This process is repeated with smaller-sized sponges as the defect contracts. Besides wound contraction, EVAC also facilitates drainage [4].

This case is an example of a complication where a multidisciplinary approach to a difficult leak following gastric sleeve to Roux-en-Y gastric bypass resulted in recovery with the use of EVAC.

This article was previously presented as a meeting abstract at the 2023 ASMBS Annual Conference in Las Vegas on June 28, 2023.

## Case presentation

We present a case of a 67-year-old woman who presented to our hospital in a semi-urban town in West Virginia, USA following a Roux-en-Y gastric bypass with hiatal hernia repair. She has a history of a gastric sleeve resection by a different surgeon, which resulted in over 50% excess body weight loss. Subsequently, she had severe gastroesophageal reflux disease, which was refractory to maximal medical therapy, which included twice-a-day high-dose proton pump inhibitors and lifestyle modifications. 

She was seen at the bariatric surgery clinic five years after her index surgery, where her medications were reviewed and patient was found to be compliant with the same. Given the refractory reflux disease, she underwent a Roux-en-Y gastric bypass with hiatal hernia repair at our hospital. At the time of this procedure, the patient weighed 91kg, with a BMI of 30kg/m2. Roux-en-Y gastric bypass surgery was performed in a standard antecolic fashion. Hiatal hernia was repaired primarily by approximating the crura of the diaphragm using non-absorbable sutures after adequate circumferential mediastinal dissection around the esophagus to increase intra-abdominal length.

The procedure and perioperative period were uneventful, and she was discharged on a bariatric stage 2 diet which includes sugar-free full liquids (focusing on sugar-free protein shakes). However, beginning on postoperative day five, she began to experience nausea, vomiting, and inability to tolerate any oral intake.

**Figure 1:**
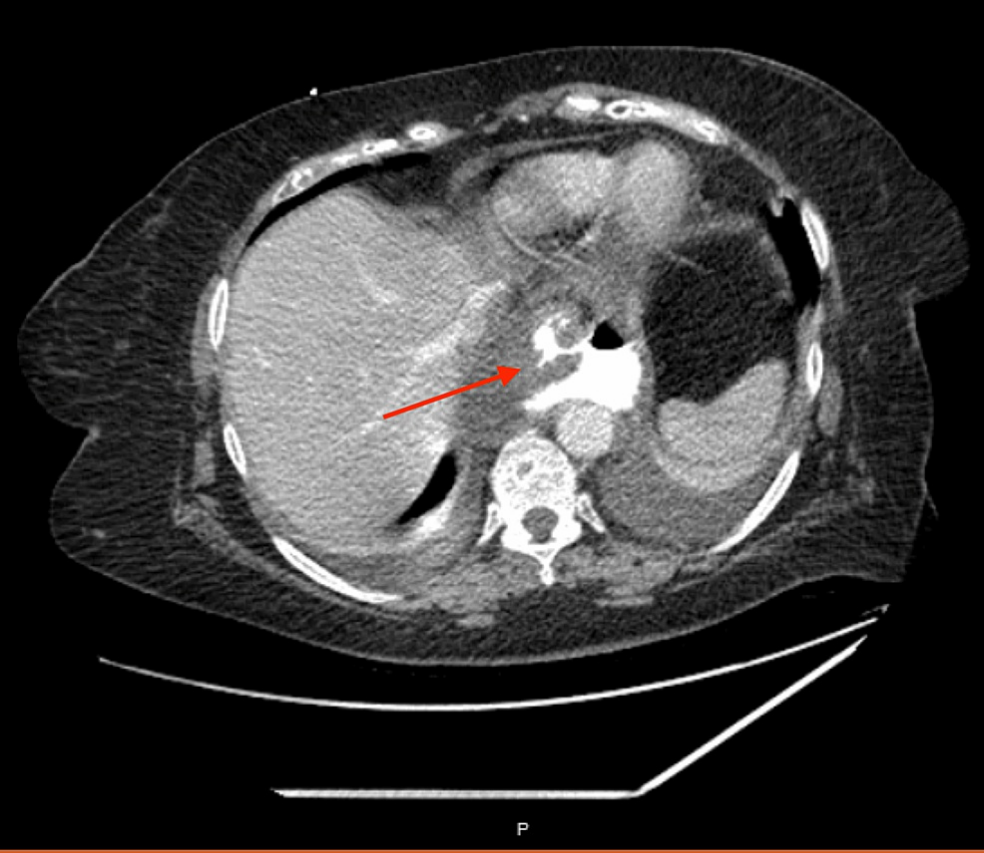
Initial CT scan revealing recurrence of retro-cardiac hernia and extravasation of oral contrast Arrow points towards the retrocardiac hernia and extravasation of oral contrast

On presentation to the Emergency Department, a CT scan was obtained (See Figure 1), which revealed a large retrocardiac hiatal hernia and bilateral pleural effusions. She was found to have leukocytosis (white blood cell count of 18000 cells/microliter) and was hemodynamically stable, but obviously dehydrated with acute kidney injury. Once in the hospital, the patient was placed on twice-a-day protonix administered intravenously, placed on antibiotics (piperacillin-tazobactam), and multimodal pain control. 

After appropriate resuscitation, she was taken to the operating room for exploration and hiatal hernia repair on the next day.

**Figure 2:**
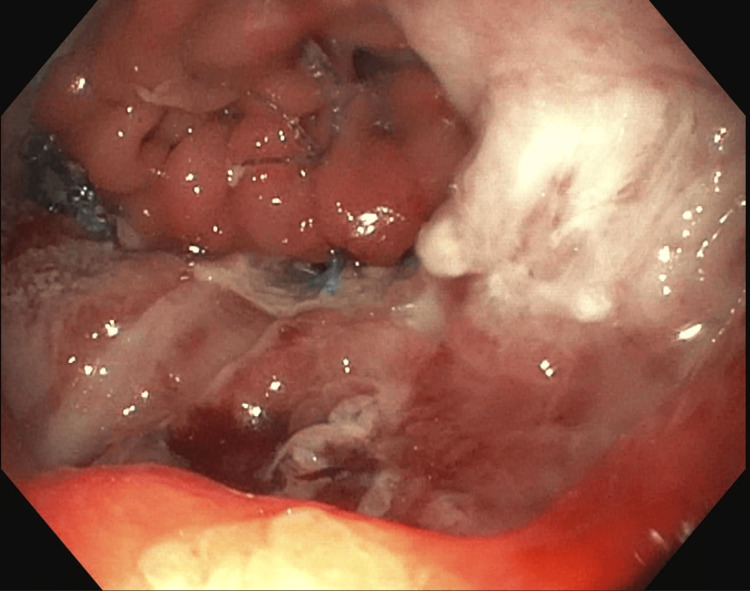
Disrupted anastomosis noted on intraoperative EGD with severely inflamed tissue EGD: esophagogastroscopy

Intraoperatively, the gastric pouch and roux limb were found to have significant edema with recurrence of the hernia. This was able to be reduced and a frank perforation was found at the posterior aspect of the anastomosis. This was carefully reduced into the abdomen, with immediate expression of large volume of purulent material from the mediastinum. A perforation was found on the posterior aspect of the pouch at the gastrojejunostomy. An intraoperative esophagogastroscopy (EGD) was performed by gastroenterology (See Figure 2), which revealed disrupted staple lines at the anastomosis. A covered metal stent was placed. A naso-jejunal tube was also placed to provide feeding access down the line. Drains were placed around the anastomosis.

The patient was transferred to the ICU. Nasojejunal tube feeds were discontinued due to suspicion for a persistent leak. The patient was placed on total parenteral nutrition (TPN) and we discussed our options with the patient and our gastroenterology colleagues. This change in plan resulted from tube feeds being noticed in the JP drains placed at the gastrojejunostomy anastomosis.

**Figure 3:**
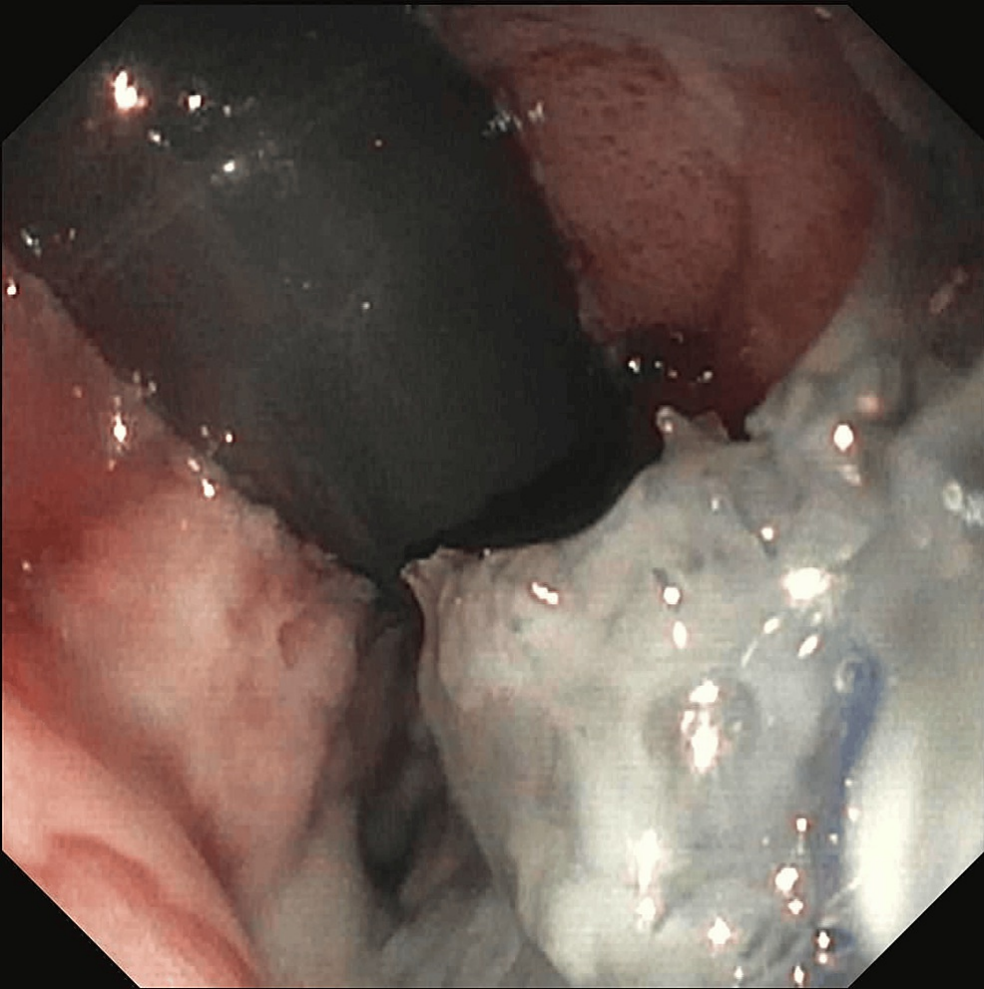
EVAC system with sponge placed at the anastomosis EVAC: endoscopic vacuum-assisted wound closure

A decision was made to place an EVAC device. The patient was taken to the gastroenterology suite and EVAC was placed and connected to vacuum via a nasogastric tube (see Figure 3). This was changed three more times with the perforation appearing better at each change. Eventually, EVAC was removed, a covered metal stent was placed, and a diet started. The patient tolerated the diet and TPN was weaned off. The patient was discharged on a stage 2 diet and has been in the clinic since. She is doing well, reports that her reflux is better, and has been tolerating a diet.

## Discussion

Anastomotic leaks are a dreaded complication following bariatric surgery. Hutter et al. in their report comparing different modalities of bariatric surgery studied data from 28,616 patients and showed a leak rate of 0.74% after sleeve gastrectomy versus 0.78% following gastric bypass [5]. Compared with patients who did not leak, Almahmeed et al. showed those with anastomotic leaks had a higher mortality (14% vs 4%, P=.01), duration of hospital stay (24.5 vs 4.5 days, P< 0.001), and overall complication rate (61% vs 20%, P < 0.001) [6]. A meta-analysis by Sajid et al. comments on staple line reinforcement decreasing the risk of anastomotic leaks post LGBP. However, they acknowledge that their findings need further analysis [7]. Given the lack of a foolproof method to prevent leaks, it then becomes necessary to effectively treat anastomotic leaks when they are discovered.

Increased pain, nausea, tachycardia, fever, and leukocytosis are suggestive of an anastomotic leak. This can be further evaluated using imaging modalities such as an upper gastrointestinal contrast study or computed tomography with intravenous and oral contrast. Once a leak is confirmed operative or nonoperative interventions can be adopted for prompt management.

Some studies have focussed on operative management of anastomotic leaks, such as the one by Jacobsen et al. studying 6030 patients. They found a leak rate of 1.1% and operatively managed 70% of them. Early leaks were treated with irrigation of the peritoneal cavity and suturing of the leak, whereas late leaks were treated with drainage. Median length of stay was 11.3 days in the patients with leaks [8].

On the other hand, analysis of a program that emphasized early detection, routine drainage, and selective nonoperative management, revealed an overall leak rate of 1.7% found on routine upper gastrointestinal series (UGI), on delayed UGI, abnormal drainage from drain, or clinical suspicion. Nonoperative management entailed restricting oral intake, antibiotics, parenteral or enteral feeding via gastrostomy or jejunostomy tubes. 71.7% of the patients were managed nonoperatively with a median hospital stay of five days versus nine days in the operative group [9]. Additionally, it would be prudent to mention that the ease of and outcome from surgical reintervention is greatly limited by highly inflamed tissue at the site of the leak [10].

With the advent and evolution of advanced gastroenterology, multiple endoscopic modalities have proven useful in managing anastomotic leaks post bariatric surgery. In a paper outlining the factors promoting successful endoscopic management of leaks following laparoscopic gastric sleeves, Ward et al. commented that a shorter duration from sentinel bariatric procedure, hemodynamically stable patient, and fewer prior bariatric interventions supported positive outcomes following endoscopic interventions. They found that patients who underwent surgical reintervention had a higher readmission rate following discharge and a higher total length of stay [11]. This could potentially be extrapolated to leaks following gastric bypass. Endoscopic application of fibrin sealant, stent placement, and endoluminal wound vacuum are methods that have been studied [4,12,13]. Endoscopic stent placement used to and often still is the modality of choice with the primary complication being stent migration. This has been shown to be as high as 40% [14]. 

EVAC consists of endoscopic debridement and washout, measurement of the cavity resulting from the leak, placement of endosponge, and connecting it to continuous suction at 175 mm Hg via a nasogastric tube. This process is repeated with smaller-sized sponges as the defect contracts. Besides wound contraction, EVAC also facilitates drainage [4]. Brangewitz et al. showed higher closure rates with EVAC at 84.4% versus 53.8% in the stent group in patients with esophageal leak. Interestingly, the EVAC group had a lower rate of strictures at 9.8% vs 28.2% [15]. At another center, EVAC had 100% closure rate for esophageal leak and 86% for gastric leaks [16]. 

While advanced endoscopic skills are essential to adequately debride the wound and place the endosponge, the promising outcomes are resulting in increased interest in this modality. In one study, the time to proficiency in EVAC for advanced endoscopists is approximately 10 cases [4].

## Conclusions

The case is a prime example of a complicated situation that could be resolved with a multidisciplinary approach involving the bariatric surgery, critical care, and advanced gastroenterology teams. In the setting of acute inflammation from perforation, where a resection of the anastomosis and reconstruction would be challenging, an endoluminal vacuum system is a viable management option.

With the morbidity associated with anastomotic leaks following gastric bypass in mind, andthe advantages of EVAC and other endoscopic modalities, it is essential that an interdisciplinary approach is used to address leaks. EVAC is a promising option in difficult anastomotic leaks post Roux-en-Y gastric bypass.
